# The Positive Effect of Authoritarian Leadership on Employee Performance: The Moderating Role of Power Distance

**DOI:** 10.3389/fpsyg.2018.00357

**Published:** 2018-03-23

**Authors:** Honglei Wang, Bichen Guan

**Affiliations:** ^1^College of Economics and Management, Northeast Agricultural University, Harbin, China; ^2^Department of Marketing and Management, Faculty of Business and Economics, Macquarie University, Sydney, NSW, Australia

**Keywords:** authoritarian leadership, learning goal orientation, power distance, employee performance, goal setting theory

## Abstract

Based on goal setting theory, this study explores the positive effect and influencing process of authoritarian leadership on employee performance, as well as the moderating role of individual power distance in this process. Data from 211 supervisor-subordinate dyads in Chinese organizations indicates that authoritarian leadership is positively associated with employee performance, and learning goal orientation mediates this relationship. Furthermore, power distance moderates the effect of authoritarian leadership on learning goal orientation, such that the effect was stronger when individual power distance was higher. The indirect effect of authoritarian leadership on employee performance via learning goal orientation is also moderated by power distance. Theoretical and managerial implications and future directions are also discussed.

## Introduction

Authoritarian leadership refers to a leader’s behavior of asserting strong authority and control over subordinates and demanding unquestioned obedience from them (Farh and Cheng, 2000). According to the leadership literature (Wang et al., 2013; Zhang and Xie, 2017), leaders who are highly on authoritarian demand their subordinates to achieve best performance among the organizations and make all the important decisions in their team. Authoritarian leadership is prevalent in Latin America, Middle East, and Asia Pacific business organizations (Pellegrini and Scandura, 2008), which has been receiving increasing attention in recent years (Schaubroeck et al., 2017). Extensive research has depicted authoritarian leadership as destructive by verifying its negative influence on employee outcomes, such as employee voice behavior (Li and Sun, 2015), team identification (Cheng and Wang, 2015), and job performance (Chan et al., 2013). Thus, high authoritarian leadership has often been considered undesirable and ineffective in organizational management.

However, some scholars have questioned the belief that authoritarian leadership is uniformly detrimental for employees and organizations, instead suggesting that it may exert positive effect on employees. For example, based on an empirical study conducted in Taiwan, Cheng et al. (2004) found authoritarian leadership to be conducive to employee responses. Tian and Sanchez’s (2017) findings suggested that authoritarian leadership was positively correlated with affective trust. Other studies have also shown weakly negative or even positive relationship between authoritarian leadership and employee performance (Farh and Cheng, 2000; Cheng et al., 2003). Such complex research findings have prompted calls for further investigation of the psychological mechanisms underlying authoritarian leadership’s effect on employee outcomes, in addition to its boundary conditions (Farh et al., 2008; Chen X.P. et al., 2014).

The mixed findings regarding the relationship between authoritarian leadership and employee outcomes suggest two possible explanations. First, the psychological processes of authoritarian leadership’s influence on employee outcomes are complex. The extant mechanisms used to explore the relationship fail to capture the full picture of the actual effect of authoritarian leadership (Cheng et al., 2004). Second, as the majority of studies on authoritarian leadership have supported its negative impact on employee behaviors, it is plausible that the actual effects of authoritarian leadership on employees depend on certain conditions, such as individual values. Authoritarian leadership is proposed to have under certain conditions a positive effect on employees. A more detailed examination of the boundary conditions may help to explain why authoritarian leadership has varying influences on employees.

To advance this line of research, we take a subordinate-centered perspective to explore the psychological process that links authoritarian leadership to employee performance, as well as the situational factor that may temper this process. From this perspective, we can gain a better understanding of how leadership shapes employee outcomes through subordinates’ self-construction. Specifically, we propose that authoritarian leadership can be positively associated with employee performance by affecting employee’s learning goal orientation. Although learning goal orientation has been considered as an individual difference in several studies (Porter, 2008), research has provided evidence that learning goal orientation can indeed be both a state and a trait, which could be enhanced by work context (Payne et al., 2007). We further argue that the relationship between authoritarian leadership and learning goal orientation is moderated by employees’ beliefs about the degree to which power should be unequally distributed in the organization (power distance). Thus, we develop and test the mediating role of learning goal orientation and the moderating role of power distance between authoritarian leadership and employee performance from a subordinate-centered perspective.

We, thereby, extend the research on authoritarian leadership in several respects. First, we discuss the possibility that authoritarian leadership may exert a positive influence on employees in the context of Chinese culture. In some Asian countries, such as China, authoritarian leadership is considered as a prevalent and effective leadership style because of its fit with traditional values (Cheng et al., 2004). Thus, it is reasonable to hypothesize that authoritarian leadership may generate a positive effect on employee performance in Chinese organizations. Second, this study deepens our understanding of the relationship between authoritarian leadership and employee performance by taking a subordinate-centered perspective. Previous studies have mainly focused on the leader-centered perspective, examining how leadership affects followers’ attitudes toward their leaders, instead of how leadership influence subordinates’ self-construction (Chan et al., 2013). Our study extends this line of research by considering the mediating role of employees’ learning goal orientation. Third, we address the inconsistent prior findings on the effects of authoritarian leadership on employee behaviors by testing power distance as a moderator. The theoretical model allows us to answer the questions of why and for whom authoritarian leadership is beneficial.

### Theory and Hypotheses

#### Authoritarian Leadership and Employee Performance

Authoritarian leadership stems from the cultural traditions of Confucianism and Legalism (Farh and Cheng, 2000; Farh et al., 2008). Under the influence of the Confucian value system, a father has absolute authority and power over his children and other family members in a traditional Chinese family (Cheng and Wang, 2015). In Chinese organizations, leaders often implement this value by establishing a centralized hierarchy and by assuming a father-like role with an authoritative leadership style (Peng et al., 2001). Thus, authoritarian leaders possesses authority over their subordinates which further induces employee compliance and submission. Also, authoritarian leaders insist on adherence to high standards and punish employees for poor performance (Wang et al., 2013). Some scholars argued that authority based on hierarchical difference predicts negative outcomes, including fear of leader, work pressure, and turnover intention (Farh and Cheng, 2000; Wang et al., 2016). However, several recent studies have also found the positive influence of authoritarian leadership on employee behaviors (Schaubroeck et al., 2017; Tian and Sanchez, 2017). Thus, results regarding whether authoritarian leadership foster or harm employee performance remain inconclusive, which calls for deeper studies exploring the relationship between authoritarian leadership and employee performance.

In our research, we propose that authoritarian leadership would enhance employee performance based on the following reasons. First, authoritarian leaders can be effective by setting specific and unambiguous goals to their subordinates. Authoritarian leaders always have the last say in their organizations and provide a singular mission upon which followers can focus on their job responsibilities, without uncertainty (Cheng et al., 2000; Schaubroeck et al., 2017). According to goal setting theory, higher performance levels are usually reached when goals are specific, rather than ambiguous (Locke and Latham, 2006). As Locke and Latham (2006) noted, when a specific goal is set for employees, goal attainment provides them with an objective, unambiguous basis for evaluating the effectiveness of their performance. Thus, although authoritarian leaders exercise tight control and unquestioned submission, the underlying reason is to promote followers’ performance.

Second, authoritarian leaders typically enhance followers’ sense of identity as group members, which further motivates employees to perform at a high level (Schaubroeck et al., 2017). As Rast et al. (2013) argued, authoritative leaders are more likely to provide a clear, unambiguous, and direct prototype with their subordinates. They usually require subordinates to obey their rules completely and punish them if they do not follow their orders (Chan et al., 2013). As a result, employees could gain a better understanding of what they should do and should not do as a team member. Prior research also suggested that authoritarian leaders offer a better sense of what it means in terms of identity, attitudes and behavior to be a member of the team (Rast et al., 2013; Schaubroeck et al., 2017). Authoritarian leaders are uniquely effective in this respect since they offer an unambiguous identity for their team members (Rast, 2015). Taking on this identity is likely to encourage an employee to dedicate effort to enhancing their performance.

Third, some scholars believe that authoritarian leaders usually set high performance standard expectations for their subordinates (Aycan, 2006). As Chen et al. (2017) argued, authoritarian leaders demand their subordinates to achieve the best performance by exercising strict control, setting clear rules, establishing job responsibilities, issuing punishment and rewards. Consequently, employees are motivated to perform strongly, delivering excellent quality. Huang et al. (2015) also claimed that authoritarian leaders, who emphasize discipline, obedience, and unity, are likely to achieve operational performance by fostering a highly centralized decision-making structure. Therefore, we expect to observe a positive relationship between authoritarian leadership and employee performance.

Hypothesis 1: Authoritarian leadership will be positively related to employee performance.

#### The Mediating Role of Learning Goal Orientation

Prior research has generally focused on the leader-centered perspective, aiming to understand the influence of authoritarian leadership behavior on subordinate by exploring how leaders affect employees’ perception of leadership behavior, such as affective trust in leader (Chen X.P. et al., 2014), team identification (Cheng and Wang, 2015), and interactive justice (Wu et al., 2012). However, most scholars have overlooked how authoritarian leadership influences employees’ self-construction, which in turn influences their reactions. The subordinate-centered perspective is important for understanding the salient impact of leadership on employee performance, as self-construction is the key motivational mechanism driving employees’ effort investment (Bono and Judge, 2003; Chan et al., 2013). We therefore explore the relationship between authoritarian leadership and employee performance from a subordinate-centered perspective to gain deeper understanding of employees’ reactions to authoritarian leadership behavior.

In our study, we propose learning goal orientation as an important mediator of authoritarian leadership’s impact on employee behavior. Achievement goal theory suggests that an individual’s goal orientation affects how he or she interprets and responds to situations and challenges (Poortvliet et al., 2007). Individuals with a strong learning goal orientation consistently strive toward mastery of a skill or task in an effort to increase competence, whereas performance goals motivate individuals to seek to gain favorable judgments of their competence or avoid negative judgments of their competence (Nicholls, 1984). Scholars have suggested that goal orientations are independent constructs, which allows an individual to possess both the learning goal and performance goal orientation simultaneously (Button et al., 1996; Anderson and Lawton, 2009).

We believe that authoritarian leadership may strengthen subordinate’s learning goal orientation. First, authoritarian leaders are highly competitive and set very high expectations for their teams (Wang et al., 2013; Zhang and Xie, 2017). Employees may realize that the best way to meet the high-level goal is not only to work hard but also work to learn and build up their competence. Also, authoritarian leaders emphasize that their team members must have the best performance of all the teams in the organization (Cheng et al., 2000). They will spread the information of achieving the best performance among the organization. Subordinates have to achieve their leaders’ high performance standards; otherwise they will be punished. These high performance standards serve as signals of insufficient goal progress, which stimulates greater effort. When employees identified the gap between their performance and their leaders’ expectation, they will build up their competence and pursue self-development through acquiring skills and task (Gong et al., 2017). Second, research shows that employees are attracted to the certainty and strength provided by authoritarian leaders and so they want to live up to their high standards. This requires continually learning and building competence. For instance, using survey data from the United Kingdom, Rast et al. (2013) found that under high uncertainty the more authoritative the leaders the more strongly their subordinates supported and trusted. Schaubroeck et al. (2017) also stated that authoritarian leaders provide unambiguous goals with which individuals can identify and ameliorate uncertainties. When employees understand what it means in terms of competences and behaviors to be a team member, they are more likely to focus on increasing their skills and striving to be suitably qualified for their work (Hogg and Adelman, 2013).

Goal orientation further influences how employees approach, interpret, and respond to situations and challenges (Dweck, 2000; Chen and Mathieu, 2008). Drawing from achievement goal theory, individuals with a strong learning goal orientation tend to pursue goals of competence improvement, which result in higher performance levels (Dweck and Leggett, 1988). Additionally, learning goal orientation is favorably related to variables involving effective self-regulation strategies and greater on-task attention (Payne et al., 2007). Prior studies consistently report positive relationships between learning goal orientation and employee performance. For example, in Taing et al.’s (2013) research, learning goal orientation was found to be associated with both setting higher goals and maintaining higher performance over time. In another study, Heslin and Latham (2004) also found learning goal orientation to be significantly associated with managerial performance. Consistent with these theoretical arguments and empirical findings, we predict the following:

Hypothesis 2: Learning goal orientation will mediate the relationship between authoritarian leadership and employee performance.

#### The Moderating Role of Power Distance

Despite the positive effect that authoritarian leaders can exert on subordinates, there are also negative aspects of authoritarianism to which many employees may respond passively (Chen X.P. et al., 2014; Li and Sun, 2015). The inconsistent effects of authoritarian leadership may be caused by the interaction of individual cultural values and leadership behaviors (Farh et al., 1997; Chen and Farh, 2010). In this section, we propose our hypothesis concerning the boundary conditions for authoritarian leadership’s positive effect, with power distance as a moderator. Power distance is the extent to which one accepts the legitimacy of unequally distributed power in institutions and organizations (Hofstede, 1980). Employees who believe that leaders should have a great degree of authority over subordinates are considered to have a high power distance, whereas employees who believe a smaller degree of authority is appropriate are considered to have a low power distance (Lee et al., 2000). Individual values on the power distance between leaders and subordinates may shape the nature of employees’ relationship with leaders (Lee et al., 2000). Thus, it is plausible to conclude that power distance may influence how individuals perceive and react to authoritarian leadership.

Employees higher in power distance have a greater psychological dependence on their leaders to offer clear goals and to establish group boundaries (Cole et al., 2013). Higher power distance individuals are more inclined to legitimize the power differences between superiors and subordinates, and develop formal and less personalized relationships with their leaders (Tyler et al., 2000). Therefore, they believe that they should not challenge their leaders and are more likely to be submissive and receptive to authoritarian leaders. Furthermore, as Schaubroeck et al. (2017) stated, subordinates higher in power distance are less likely to expect to be consulted by or receive information from authoritarian leaders about their work. Having great respect for authority, they interpret authoritative behaviors as more favorable than passive behaviors, and prefer superiors who exhibit authoritarianism (Hofstede, 1980). For example, Chen C.C. et al. (2014) argued that high power distance employees perceive standard setting and management control as signs of consideration and support, rather than undue interference. Consequently, these subordinates are more likely to focus on their jobs and be motivated to enhance their competences and abilities.

Conversely, employees with lower power distance are more likely to expect and develop personalized relationships with their leaders, as they view leaders as approachable (Hofstede, 1980; Tyler et al., 2000). Such individuals care more strongly about how they are treated by authority figures who oversee their work (Lian et al., 2012). Given that lower power distance subordinates expect superiors to consult them and value their opinions, they are inclined to react negatively when superiors seem to be authoritative. Compared with higher power distance employees, employees expecting to be strongly connected to authority figures may feel disrespected and unduly controlled when leaders exhibit authoritarianism. As such, lower power distance subordinates are more likely to experience the negative influence of authoritarian leadership; thus, the positive effect of authoritarian leadership on individuals’ learning goal orientation may be reduced for employees with lower power distance.

Hypothesis 3: Power distance will moderate the relationship between authoritarian leadership and learning goal orientation, such that when power distance was higher, the positive effect of authoritarian leadership on learning goal orientation was higher; when power distance was lower, the positive effect of authoritarian leadership on learning goal orientation was lower.

**FIGURE 1:**
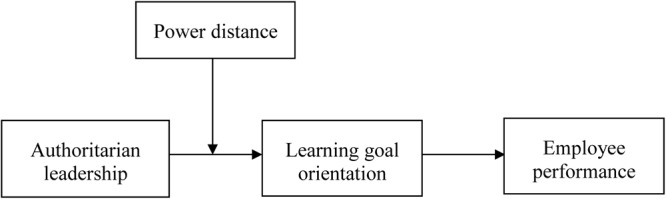
Theoretical model.

We further propose that power distance will moderate the indirect relationship of authoritarian leadership on employee performance through learning goal orientation. Thus, we develop a moderated mediation hypothesis and build up our theoretical model. **Figure 1** illustrates the study’s theoretical model.

## Materials and Methods

### Sample and Procedure

The study’s sample comprises 211 supervisor-subordinate dyads from 10 different technology companies located in China. To avoid common method bias, the data were sourced from multiple independent teams and from multiple respondents within each team. Surveys were distributed to potential participants through human resource management departments. Data were principally collected by surveying managers and employees within each team. The respondents were assured of confidentiality and that nobody else in their teams would have access to their individual responses. To maximize the response rate, managers were contacted through a follow-up phone call or email 2 weeks after the initial distribution of the survey. Out of 280 distributed questionnaires (40 to supervisors and 240 to subordinates), 260 questionnaires (representing 232 supervisor-subordinate dyads) were returned, giving a response rate of 92.8% for both leaders and subordinates. 21 pairs of responses were deleted as either they did not provide data on key variables or showed obvious random responding (Osborne and Blanchard, 2011) (e.g., in a survey utilizing a Likert scale, the respondent only give answers as 1’s or 5’s). These omissions resulted in a usable sample of 211 supervisor-subordinate dyads. In the employee sample, 36.5% were male, 4.0% were aged 25 or younger, 63.5% were aged between 26 and 40, 32.5% were aged 41 or older, and 86.4% of the employee respondents have received at least a college education.

### Measures

Except for the items on authoritarian leadership, all the measures used in this study were adopted from English literature. In accordance with (Brislin et al.’s (1973) back-translation procedure, the primary researcher (a native Chinese speaker who is also proficient in English) translated the measures from English into Chinese. Next, the primary researcher and another researcher (with human resource management experience in the Chinese workplace) both checked the translation for accuracy, identified problematic areas, and improved the translation through an iterative process. Finally, the translation was validated by a Ph.D. student (a Chinese native with a doctorate from the United States), who improved the readability of the questions through discussion with the other two researchers. All measures used five-point Likert-type response categories (ranging from 1 “strongly disagree” to 5 “strongly agree”).

#### Authoritarian Leadership

To measure authoritarian leadership, we used nine-item scale developed by Cheng et al. (2004). Sample items for authoritarian leadership were: “My supervisor determines all decisions in the organization whether they are important or not” and “My supervisor emphasizes that our group must have the best performance of all the units in the organization.” The Cronbach’s alpha coefficient for the measure of authoritarian leadership was 0.83.

#### Learning Goal Orientation

Learning goal orientation was assessed using nine items scale developed by VandeWalle (1997). Two sample items were “I often read materials related to my work to improve my ability,” and “I often look for opportunities to develop new skills and knowledge.” The Cronbach’ s alpha coefficient was 0.88.

#### Power Distance

We measured power distance using a six-item measure developed by Dorfman and Howell (1988) for use in Taiwan. Sample items were “Managers should make most decisions without consulting subordinates” and “Employees should not disagree with management decisions.” In this study, the coefficient alpha for the measure of power distance was 0.86.

#### Employee Performance

The team managers were asked to provide a performance rating for each individual employee. We used three items from a scale developed by Heilman et al. (1992). Sample items were “This employee is very competent,” “This employee gets his or her work done very effectively,” and “This employee has performed his/her job well.” The Cronbach’s alpha coefficient for the measure of employee performance was 0.86.

#### Control Variables

Prior research has found that demographic variables (gender and age) may influence employee performance (Shore et al., 2003; Schaubroeck et al., 2007), we therefore controlled for gender and age in our study. In addition, we controlled for leader-member exchange as it has shown a positive relationship with employee performance (Dulebohn et al., 2012; Martin et al., 2016). Gender was coded 0 for “female” and 1 for “male.” Age was measured by number of years. Leader-member exchange was measured using seven items scale developed by Scandura and Graen (1984). The Cronbach’ s alpha coefficient of the scale was 0.87.

## Results

### Measurement Validation

We conducted confirmatory factor analyses (CFA) in Mplus 7 to test the distinctiveness of the variables included in the study: authoritarian leadership, learning goal orientation, power distance, and employee performance. As indicated in **Table 1**, the hypothesized four-factor model fits the data well, χ^2^(*df* = 306) = 561.69, root-mean-square error of approximation (RMSEA) = 0.06, standardized root mean square residual (SRMR) = 0.08, comparative fit index (CFI) = 0.91. Against this baseline model, we test three alternative models: a three-factor model combining authoritarian leadership and learning goal orientation into one factor; a two-factor model combining authoritarian leadership, learning goal orientation, and power distance into one factor; and a single-factor model combining all four variables into one factor. As shown in **Table 1**, the baseline model fits the data significantly better than all three alternative models, indicating that the four variables show good discriminant validity. Thus, we retained the hypothesized four-factor model for our analyses. Then, we tested for common method variance (CMV) with a CFA model wherein all the items loaded on the respective factors and a common method factor (Podsakoff et al., 2003). The average variance explained by the common method factor was 21%, less than 25%, the median reported by Williams et al. (1989).

**Table 1:** Comparison of factor structures.

Model	χ^2^*(df)*	Δχ^2^*(df)*	RMSEA	SRMR	CFI
The hypothesized four-factor model	561.69 (306)		0.06	0.08	0.91
A three-factor model combining authoritarian leadership and learning goal orientation	973.65 (314)	411.96 (8)	0.10	0.15	0.76
A two-factor model combining authoritarian leadership, learning goal orientation, and power distance	1206.62 (315)	644.93 (9)	0.09	0.13	0.67
A single-factor model combining all four variables	1332.86 (315)	771.17 (9)	0.12	0.16	0.62

*N = 211. The second, third, and fourth models were compared with the first (hypothesized four-factor) model.*

Also, based on the work of Williams et al. (2010), we applied the CFA marker technique to further examine the CMV in our data. This method uses a marker variable that is theoretically unrelated to the substantive variables in the proposed model to test the CMV. We selected hindrance stressor (Cavanaugh et al., 2000) as a marker variable since it showed the weakest correlation with other variables (**Table 2**). Hindrance stressor was measured with five items and sample items were “The amount of red tape I need to go through to get my job done” and “The degree to which politics rather than performance affects organizational decisions.” According to the procedure of the CFA marker technique, we analyzed the CFA model, baseline model, Method-C model, Method-R model, and Method-U model. The results indicated that the Method-R model was not superior to the Method-U model (Δχ^2^ = 11.9, *df* = 16, *p* > 0.75). Therefore, there is no severe CMV in our study.

**Table 2:** Means, standard deviations, and correlations among all variables.

	Mean	*SD*	1	2	3	4	5	6	7
(1) Gender	1.67	0.53							
(2) Age	2.29	0.54	0.07						
(3) LMX	4.13	0.73	–0.01	0.06					
(4) Authoritarian leadership	3.08	0.68	–0.24^∗∗^	0.08	0.32^∗∗^				
(5) Learning goal orientation	3.40	0.97	–0.18^∗∗^	–0.13	–0.13	0.18^∗∗^			
(6) Power distance	2.49	0.76	–0.09	0.08	0.08	0.45^∗∗^	0.16^∗^		
(7) Employee performance	4.18	0.73	–0.05	0.15	0.81^∗∗^	0.24^∗∗^	0.40^∗∗^	–0.16	
(8) Hindrance stressor	2.68	0.83	0.01	0.11	–0.11	0.07	–0.08	0.10	–0.03

*N = 211; ^∗^*p* < 0.05, ^∗∗^*p* < 0.01.*

### Descriptive Statistics

We present the means, standard deviations, and correlations among all the variables in **Table 2**. The results show that authoritarian leadership is positively related to learning goal orientation (*r* = 0.18, *p* < 0.01), power distance (*r* = 0.45, *p* < 0.01), and employee performance (*r* = 0.24, *p* < 0.01). The results also support the positive relationship between learning goal orientation and employee performance (*r* = 0.40, *p* < 0.01).

### Hypotheses Testing

**FIGURE 2:**
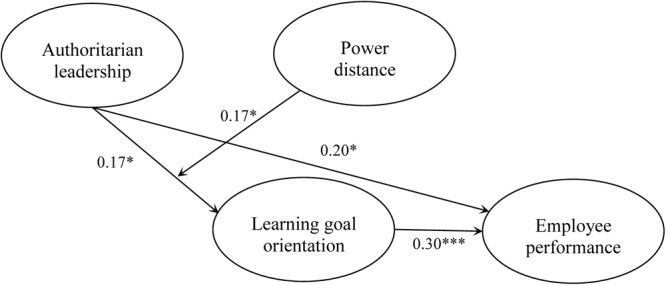
Model results. ^∗^*p* < 0.05, ^∗∗^*p* < 0.01, ^∗∗∗^*p* < 0.001.

To test the main and mediation effects, we used the path analysis model conducted in Mplus 7, which estimate both the path coefficients and the indirect effects with bootstrapping. As shown in **Figure 2**, after controlling for gender, age and leader-member exchange, authoritarian leadership has a positive relationship with learning goal orientation (*B* = 0.17, *SE* = 0.08, *p* < 0.05) and employee performance (*B* = 0.20, *SE* = 0.08, *p* < 0.05). The positive relationship between learning goal orientation and employee performance is also significant (*B* = 0.30, *SE* = 0.10, *p* < 0.001). The bootstrapping results further suggest that the indirect effect between authoritarian leadership and employee performance via learning goal orientation is significant (indirect effect = 0.05, *SE* = 0.03, 95% CI = [0.007, 0.131], excluding zero). These findings support Hypotheses 1 and 2.

**FIGURE 3:**
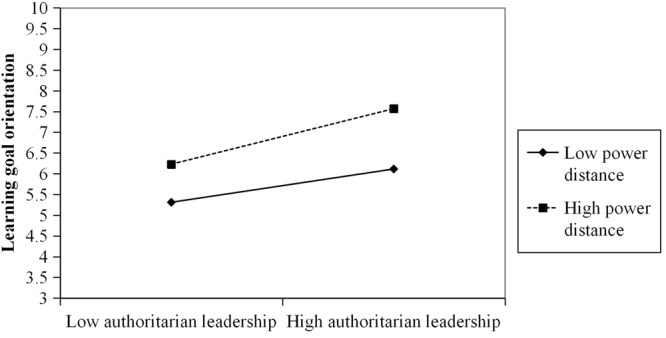
Interaction between authoritarian leadership and power distance on learning goal orientation.

Hypothesis 3 proposes the moderating effect of power distance on the relationship between authoritarian leadership and learning goal orientation. We examined this hypothesis by adding an interaction term of authoritarian leadership and power distance into the model predicting learning goal orientation. The results reveal that the predicted interaction is significant (*B* = 0.17, *SE* = 0.07, *p* < 0.05). To further interpret the nature of this significant interaction, we plotted the relationship between authoritarian leadership and learning goal orientation at 1 *SD* above and below the mean of the moderator (Aiken and West, 1991). **Figure 3** shows the moderating role of power distance: supporting our hypothesis, when individual’s power distance was higher, the positive effect of authoritarian leadership on learning goal orientation was stronger (*B* = 0.98, *t* = 2.48, *p* < 0.05). However, when individual’s power distance was lower, the positive effect of authoritarian leadership on learning goal orientation was weaker (*B* = 0.59, *t* = 2.59, *p* < 0.05). Furthermore, we examined whether power distance moderated the indirect effect of authoritarian leadership on employee performance through learning goal orientation. The findings revealed that the indirect effect was significant in the condition of higher power distance (indirect effect = 0.08, *SE* = 0.04, 95% CI = [0.03, 0.17], excluding zero), whereas the indirect effect was not significant in the condition of lower power distance (indirect effect = 0.02, *SE* = 0.04, 95% CI = [-0.03, 0.12], including zero). Therefore, the results are consistent with Hypothesis 3.

## Discussion

The primary goal of our research is to examine how, why, and under what condition authoritarian leadership may exert a positive effect on employee performance. In particular, we proposed and tested the mediating role of learning goal orientation on the relationship between authoritarian leadership and employee performance. We then examined the moderating effect of individual power distance on the impact of authoritarian leadership on learning goal orientation. Our findings reveal that authoritarian leadership is positively related to employee performance through learning goal orientation, and the strength of the relationship is dependent on employees’ power distance.

### Theoretical Implications

Authoritarian leadership is widely considered as the exemplar of detrimental leadership behaviors. Previous studies of authoritarian leadership have primarily emphasized and highlighted its negative features (Chan et al., 2013; Chen X.P. et al., 2014). However, recent studies have started to explore the potential positive influence of authoritarian leadership (Huang et al., 2015; Tian and Sanchez, 2017), suggesting that the mechanisms through which authoritarian leadership influences employee outcomes still require further investigation. Our study attempted to address the lack of consensus on whether authoritarian leadership is beneficial for or detrimental to employee performance. Our findings indicate that authoritarian leadership is positively related to employee performance (Hypothesis 1). This result is consistent with previous research findings and the argument that employees in Chinese organizations may consider authoritarian leadership behavior to be the norm and show greater tolerance for this type of leadership behavior (Cheng et al., 2000; Tian and Sanchez, 2017). Therefore, our study’s results add important evidence to the literature concerning the actual effect of authoritarian leadership on employees. In addition, despite theoretical arguments that authoritarian leadership may promote positive outcomes (Cheng et al., 2004), only a few studies have provided empirical evidence (Tian and Sanchez, 2017). In this respect, our study offers a fresh insight into the performance implications of authoritarian leadership and contributes to authoritarian leadership research.

Second, by exploring the effect of authoritarian leadership on employee performance through individuals’ learning goal orientation, we were able to obtain a richer picture of the mechanisms through which authoritarian leadership affects employees. Prior research has generally examined the effect of leadership (e.g., transformational leadership, ethical leadership) through a leader-centered approach, ignoring the role of subordinates’ self-construction, despite its verified importance in explaining the function of leadership (Chan et al., 2013; Schaubroeck et al., 2017). This study extends the scope of this approach and suggests that learning goal orientation plays a mediating role in the relationship between authoritarian leadership and employee performance. Building on goal setting theory, we argued that employees would be motivated to enhance their competence and performance under the specific and difficult goals offered by authoritarian leaders. Our research, thereby, provides more comprehensive understanding of subordinates’ role in the process of authoritarian leadership influencing employee outcomes.

Third, our findings indicate that the positive relationship between authoritarian leadership and learning goal orientation is enhanced when employees hold higher levels of power distance and mitigated when they hold lower levels. Consistent with the work of Schaubroeck et al. (2017), we argue that the difference between individuals with higher and lower power distance could simply reflect how higher power distance norms and values are associated with weaker needs for personal influence (Daniels and Greguras, 2014). Individual power distance has great implications for the ways in which authoritarian leaders are evaluated by employees. Employees with higher power distance are inclined to consider authoritarianism as reasonable and, therefore, more favorably interpret of authoritarian leadership behavior. Our work, thus, provides further evidence of the favorable role of power distance in the process of authoritarian leaders exerting influence on employees, and develops our understanding of the complex effects of authoritarianism.

### Managerial Implications

Our research also has several managerial implications. Although some studies have shown negative effects on employees who experience authoritarian leadership, managers need to be aware that authoritarian leadership may also motivate employees to enhance their performance; this is particularly the case in Chinese organizations. Indeed, some scholars have already suggested the positive effect of authoritarian leadership on firm performance when firms operate in resource–scarce environments (Huang et al., 2015). Leaders who focus on discipline and rules may motivate their subordinates to enhance their abilities and performance in Chinese organizations. We also found that the association between authoritarian leadership and employee outcomes may vary depending on individual power distance. For employees higher in power distance, authoritarian leadership could exert a more positive effect on employee performance; however, for individuals lower in power distance, the positive effect may be weaker. Therefore, it is reasonable for authoritarian leaders to assess individuals’ power distance during the selection process.

### Limitations and Future Directions

Despite some notable contributions, this study has several limitations that indicate future research avenues. First, we used a Chinese sample, which might limit the generalizability of the research findings to other cultural contexts (Pellegrini and Scandura, 2008). Since China’s culture is characterized by high power distance and collectivism (Hofstede, 2001), it is plausible that subordinates are more tolerant of authoritarian leaders than their counterparts in other cultures (Chan et al., 2013). Therefore, it would be valuable for future studies to verify our findings in different cultural contexts. Second, results based on the technology company employees we surveyed may not be generalizable to other work settings. Some research showed that specific conditions such as uncertainties (Rast et al., 2013) and low economic munificence (Huang et al., 2015) may enhance authoritarian leaders effectiveness. Thus, it is worthwhile for future research to extend the current analysis to other types of industries (e.g., manufacturing or service settings). Third, our research used a cross-sectional design and self-reported individual-level measurements of the independent, mediating, and moderating variables. We employed the latent method factor in CFA to extract the influence caused by CMV. However, future research could use longitudinal designs to examine the causal relationships and further reduce the possible influence of CMV.

In addition to these limitations, we also suggest some new directions for future research. First, future studies could build on our work by further exploring how authoritarian leadership affects other employee outcomes. For example, it would be interesting to investigate whether authoritarian leadership could benefit employees through enhancing their job focus and work engagement. Second, while we test the mediating role of learning goal orientation in the process of authoritarian leadership affecting employee outcomes, future study could expand the range of potential mediators to consider other self-related constructs, such as core self-evaluation (Kacmar et al., 2009) and self-esteem (Chan et al., 2013). Based on social identity theory, recent study has already examined the mediating role of perceived insider status between authoritarian leadership and employee outcomes (Schaubroeck et al., 2017). Third, we have made assumptions about the moderating role of individual power distance. Future research could consider including other contextual factors to help explain the inconsistent findings in authoritarian leadership literature. For example, the effect of authoritarian leaders on subordinates may be affected by leader characteristics, such as leader integrity, or situational factors, such as organizational justice.

## Ethics Statement

An ethics approval was not required as per institutional guidelines and national laws and regulations because no unethical behaviors existed in this study. We just conducted paper–pencil test and were exempt from further ethics board approval since this research did not involve human clinical trials or animal experiments. All subjects gave written informed consent in accordance with the Declaration of Helsinki. Research respondents were ensured confidentiality and anonymity. All participation was voluntary.

## Author Contributions

HW has been provided substantial contribution to the research design, data collection, and the write-up. BG has been involved in data analysis and interpretation. All authors reviewed and approved this paper for publication.

## Conflict of Interest Statement

The authors declare that the research was conducted in the absence of any commercial or financial relationships that could be construed as a potential conflict of interest.
